# Pur-Alpha Induces JCV Gene Expression and Viral Replication by Suppressing SRSF1 in Glial Cells

**DOI:** 10.1371/journal.pone.0156819

**Published:** 2016-06-03

**Authors:** Ilker Kudret Sariyer, Rahsan Sariyer, Jessica Otte, Jennifer Gordon

**Affiliations:** Department of Neuroscience, Center for Neurovirology, Temple University Lewis Katz School of Medicine, 3500 North Broad Street, 7th Floor, Philadelphia, PA 19140, United States of America; University of Utah, UNITED STATES

## Abstract

**Objective:**

PML is a rare and fatal demyelinating disease of the CNS caused by the human polyomavirus, JC virus (JCV), which occurs in AIDS patients and those on immunosuppressive monoclonal antibody therapies (mAbs). We sought to identify mechanisms that could stimulate reactivation of JCV in a cell culture model system and targeted pathways which could affect early gene transcription and JCV T-antigen production, which are key steps of the viral life cycle for blocking reactivation of JCV. Two important regulatory partners we have previously identified for T-antigen include Pur-alpha and SRSF1 (SF2/ASF). SRSF1, an alternative splicing factor, is a potential regulator of JCV whose overexpression in glial cells strongly suppresses viral gene expression and replication. Pur-alpha has been most extensively characterized as a sequence-specific DNA- and RNA-binding protein which directs both viral gene transcription and mRNA translation, and is a potent inducer of the JCV early promoter through binding to T-antigen.

**Methods and Results:**

Pur-alpha and SRSF1 both act directly as transcriptional regulators of the JCV promoter and here we have observed that Pur-alpha is capable of ameliorating SRSF1-mediated suppression of JCV gene expression and viral replication. Interestingly, Pur-alpha exerted its effect by suppressing SRSF1 at both the protein and mRNA levels in glial cells suggesting this effect can occur independent of T-antigen. Pur-alpha and SRSF1 were both localized to oligodendrocyte inclusion bodies by immunohistochemistry in brain sections from patients with HIV-1 associated PML. Interestingly, inclusion bodies were typically positive for either Pur-alpha or SRSF1, though some cells appeared to be positive for both proteins.

**Conclusions:**

Taken together, these results indicate the presence of an antagonistic interaction between these two proteins in regulating of JCV gene expression and viral replication and suggests that they play an important role during viral reactivation leading to development of PML.

## Introduction

Progressive multifocal leukoencephalopathy (PML) is a fatal demyelinating disease of the brain caused by the reactivation of JC virus from a low level persistent state. Upon reactivation from latency sites, JC virus traffics into the brain where the virus infects oligodendrocytes, the myelin-producing cells of the brain. JC virus replication in oligodendrocytes leads to lytic destruction of the infected cells, thus destroying myelin sheaths. Once considered a rare disease, PML first received considerable attention due to a rapid rise in incidence at the onset of the AIDS epidemic. In the last several years, attention to PML has increased significantly due to its occurrence in patients receiving natalizumab, an anti-integrin antibody therapy for MS [1–3]. PML has been reported as an adverse event in the context of treatment of other auto-immune disorders with a variety of monoclonal antibody therapies, suggesting that immunosuppression can predispose these patients to the development of PML as well. These include efalizumab (Raptiva) for the treatment of plaque psoriasis, which targets CD11a on T cells [4] and three monoclonal antibodies targeting TNF-alpha, adalimumab (Humira), etanercept (Enbrel), and infliximab (Remicade) for the treatment of psoriasis, rheumatoid arthritis, and Crohn's disease. Interestingly, rituximab (trade named Rituxan), which targets CD20 on circulating B cells and is thought to lead to their depletion, has been associated with considerable cases of PML in B cell lymphoma and rheumatoid arthritis patients [5]. There are no effective treatments for PML and it is usually fatal within 6–12 months [6]. Currently, the only option for PML patients is reduction in immunosuppression or restoration of underlying immune impairment. This is based on early observations that the availability of HAART treatment improved patient survival in HIV-1+ PML patients [7–10]. Presumably, the deficiency of CD4+ T cells seen in treatment naive HIV/AIDS patients results in a lack of JCV specific CD8+ effector T cells, thus predisposing HIV-1+ patients to the risk for PML development [11]. A broad range of drugs, some with potential anti-viral mechanisms of action have been tested and have failed at controlling JC virus replication and halting its progression in PML patients.

JCV has a double-stranded, DNA genome enclosed within an icosahedral capsid. The noncoding regulatory region acts as a bidirectional viral promoter separating the viral genome into early and late genes. The early JCV transcript encodes the major regulatory proteins, large and small T antigens (T-antigen and t-antigen, respectively). As described in detail previously [12], T-antigen is the key viral regulatory protein, which acts as a transcriptional factor to autoregulate its own viral promoter and drive the downstream steps of the viral life cycle, including DNA replication and expression of the late viral transcript, which encodes the accessory protein, agnoprotein, and capsid proteins VP1, VP2, and VP3 [13]. In driving viral replication, T-antigen hijacks the host cellular machinery, via sequestration of key cellular regulators. For example, T-antigen stabilizes key members of the Wnt signaling pathway, β-catenin and c-myc, allowing for them to more readily enter the nucleus [14–16], which impacts several genes downstream of these transcription factors, including Oct4, Nanog and Sox2, which promote self-renewal, a characteristic of stem cells. Further, T-antigen can allow infected cells to evade apoptosis through its interactions with the IGF-1 receptor, which enhances mTOR signaling and expression of the anti-apoptotic protein, surviving [17]. Alternatively, BAG3, another inhibitor of apoptosis, can suppress T-antigen expression and halt JCV replication [18]. The induction of early gene transcription by JCV T-antigen is thus the first step in viral replication and a key potential target for blocking reactivation of JCV.

Two important cellular proteins which are capable of regulating JCV gene expression and viral replication include Pur-alpha and SRSF1. We recently identified the alternative splicing factor, SRSF1, as a potential regulator of JCV whose overexpression in glial cells strongly suppresses viral gene expression and replication [19–21]. Down-regulation of JCV by SRSF1 is mediated at the step of splicing of the JCV early genes including T-antigen pre-mRNA as well as at the step of viral early gene transcription thus ascribing an important role for SRSF1 in the control of JCV gene expression. Pur-alpha has been most extensively characterized as a sequence-specific single-stranded DNA- and RNA-binding protein which directs both DNA replication and gene transcription [22,23]. In the nucleus, it associates with cellular DNA to activate or suppress transcription of a number of cellular genes including myelin basic protein, gata2, TNFα, TGFβ, and E2F1 as well as the Pur-alpha promoter itself [24] and interacts with a number of key cellular regulatory proteins including Rb, E2F1, and several cyclins and cdks [22,25,26]. In the cytoplasm, Pur-alpha plays an increasingly recognized role as a critical component in mRNA translation [21,27,28]. Pur-alpha is a potent inducer of JCV replication through direct induction of viral gene expression and binding to the key viral regulatory protein, T-antigen [22,29–31].

Given the known roles of both SRSF1 and Pur-alpha in regulating target nucleic acid sequences including those of JCV at both the DNA and RNA levels, here we analyzed possible molecular interplay between Pur-alpha and SRSF1 in regulation of JCV gene expression and viral replication. Our results have revealed an antagonistic functional interaction between Pur-alpha and SRSF1 in controlling JCV gene transcription and viral T-antigen expression that may suggest a novel mechanism for the reactivation of JCV and initiation of the JCV lytic replication cycle in glial cells.

## Results

### Pur-alpha inhibits SRSF1-mediated suppression of JCV transcription

**Fig 1:**
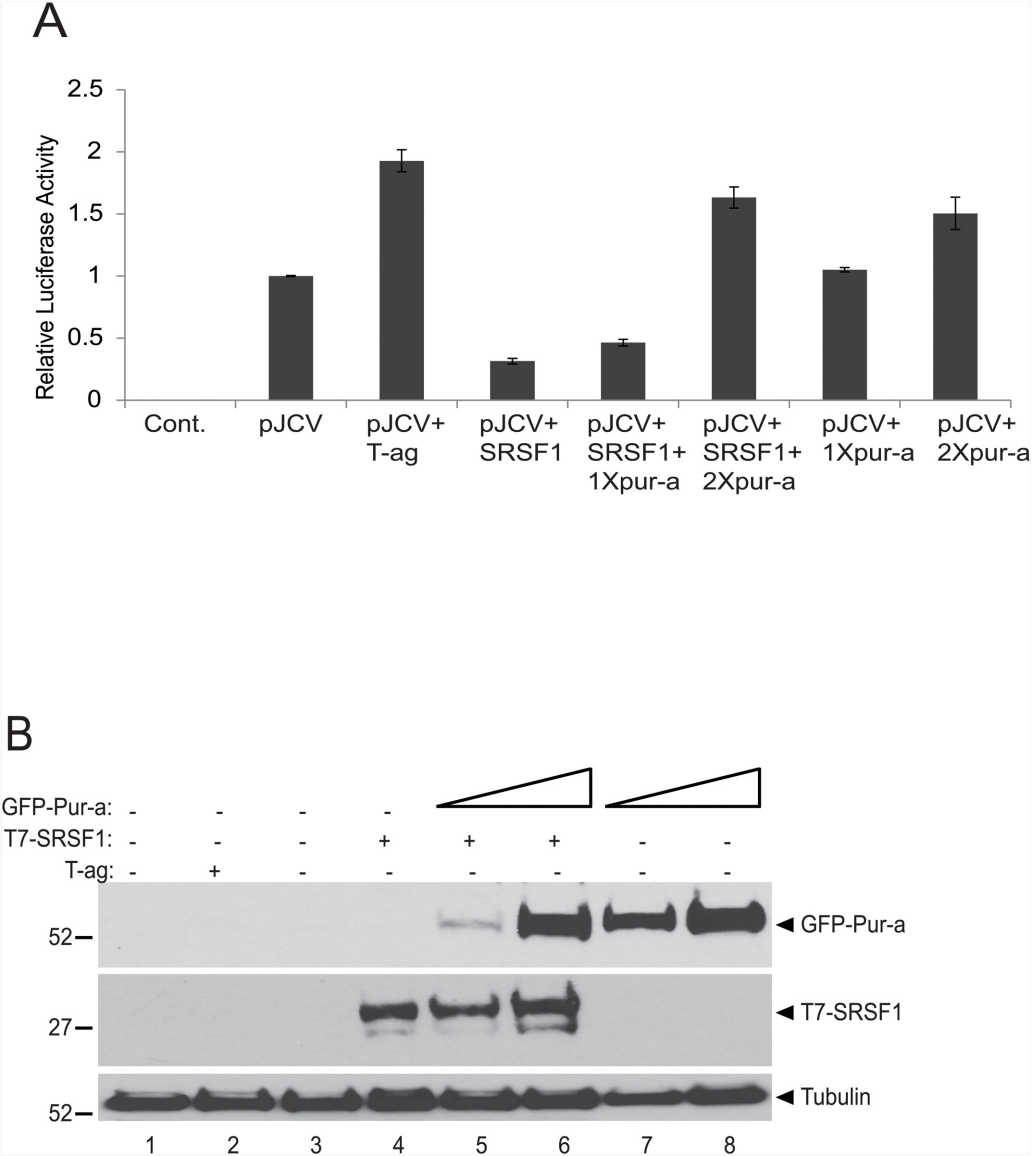
Pur-alpha blocks SRSF1-mediated transcriptional suppression of JCV promoter. A. T98G cells were transiently transfected with pGL3-JCV-early reporter construct (pJCV) expression plasmid encoding T-antigen, and increasing concentrations (1X and 2X) of plasmids encoding T7-tagged SRSF1, and GFP-tagged Pur-alpha as indicated. At 48 hr post-transfection, luciferase activities were determined and are presented as a bar graph as activity relative to pJCV plasmid alone. B. Western blot analysis of the same protein samples used in luciferase assays (panel A) for the expression of T7-tagged SRSF1 and GFP-tagged Pur-alpha. Tubulin was probed in the same blots as an internal loading control.

The ability of Pur-alpha to induce JCV reactivation has been well established by our group during the last two decades [29–31]. More recently, we have identified another cellular gene, SRSF1, which functions as a suppressor of JCV reactivation [19–21,32]. Even though they function inversely in regulation of JCV gene expression, Pur-alpha and SRSF1 share several similarities including regulation of mRNA as well as serving as transcription factors. We therefore examined the functional interplay between Pur-alpha and SRSF1 in regulating JCV early gene transcription. As shown in Fig 1, transient transfection of glial cells with T7-tagged SRSF1 led to a reduction in JCV early promoter activity as determined by l assay (panel A, lane 4). Conversely, transfection of GFP-Pur-alpha induced JCV promoter activity approximately 1.75 fold (panel A, lanes 7 and 8). This induction is nearly as high as the 2-fold activity induced by JCV T-antigen itself, which is known to upregulate the JCV early promoter and therefore was used as positive control in the assay (panel a, lane 3). Pur-alpha's effect on JCV promoter activity was even strong enough to abrogate the down regulation mediated by SRSF1 (panel a, lanes 5–6). These results suggest that Pur-alpha is capable of reversing the SRSF1-mediated suppression of JCV transcription.

### Pur-alpha ameliorates SRSF1-mediated suppression of T-antigen expression

**Fig 2:**
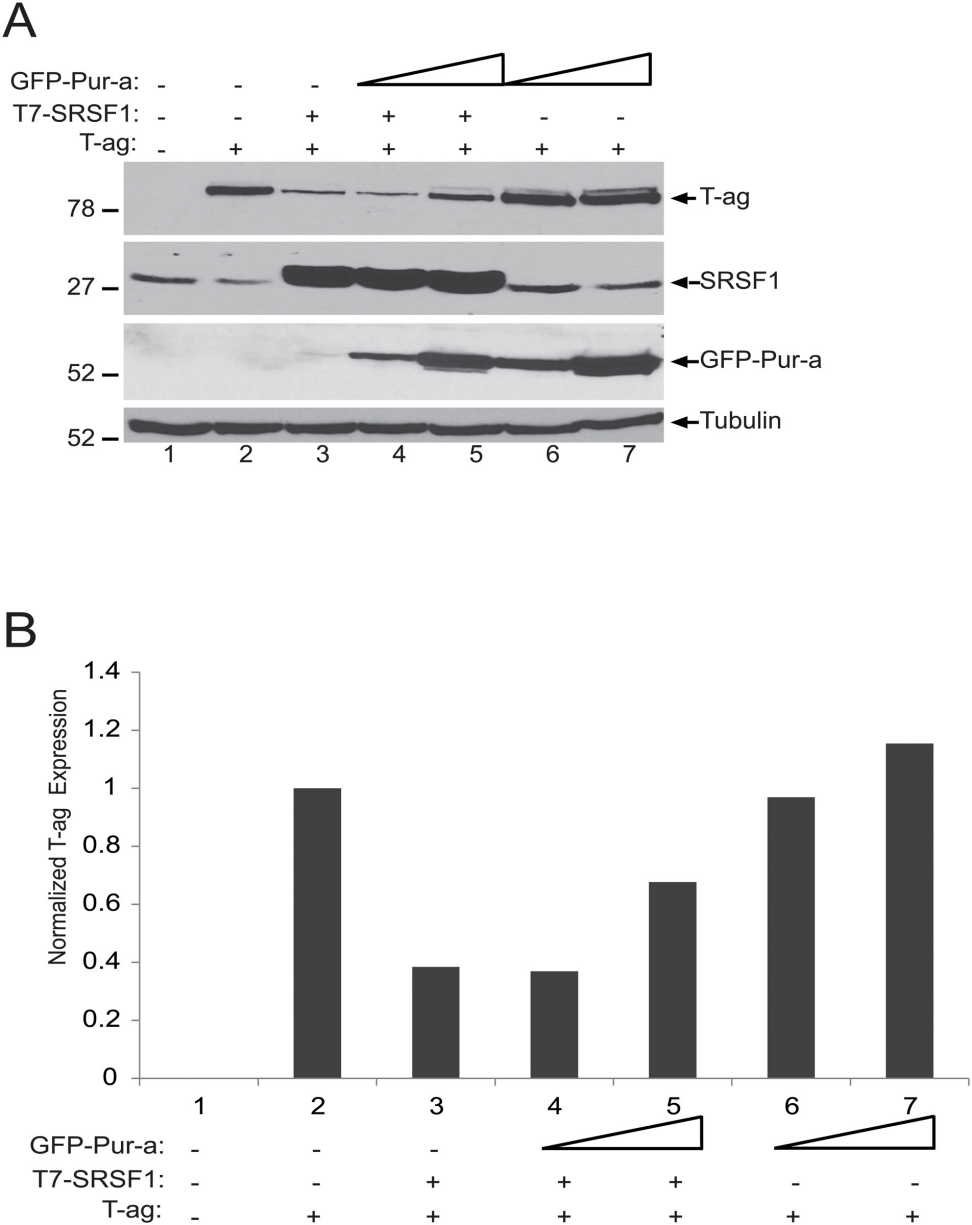
SRSF1-mediated suppression of JCV T-antigen is ameliorated by Pur-alpha. A. T98G cells were transiently transfected with expression plasmids encoding JCV T-antigen, T7-tagged SRSF1 and GFP-tagged Pur-alpha as indicated. Expression of the proteins were detected by Western blotting using anti-T-antigen, anti-SRSF1, and anti-GFP antibodies. Tubulin was probed in the same blots as an internal loading control. B. Bar graph presentation T-antigen expression levels based on signal intensity of T-antigen normalized to levels of the tubulin internal loading control in panel A.

We recently showed that SRSF1 inhibits JCV T-antigen expression in glial cells [19–32]. Our reporter assays presented in Fig 1 suggested that Pur-alpha is able to attenuate the negative suppressive effect of SRSF1 on JCV early gene transcription. We next examine the potential impact of Pur-alpha on SRSF1-mediated suppression of T-antigen expression. T98G cells were transiently transfected with expression plasmids encoding T-antigen, T7-tagged SRSF1, and increasing concentrations of GFP-tagged Pur-alpha. At 48 hrs post-transfections, whole cell protein lysates were prepared and examined by Western blotting for the detection of T-antigen, SRSF1, and GFP-Pur-alpha. As shown in Fig 2A and 2B, consistent with previous observations, SRSF1 expression in glial cells decreased the levels of T-antigen (compare lanes 2 and 3), which were partially restored by co-expression of increasing concentrations of Pur-alpha (lanes 4 and 5). These observations suggest that Pur-alpha is capable of recovering SRSF1-mediated suppression of T-antigen protein levels.

### Ectopic expression of Pur-alpha inhibits SRSF1 expression in astrocytes

**Fig 3:**
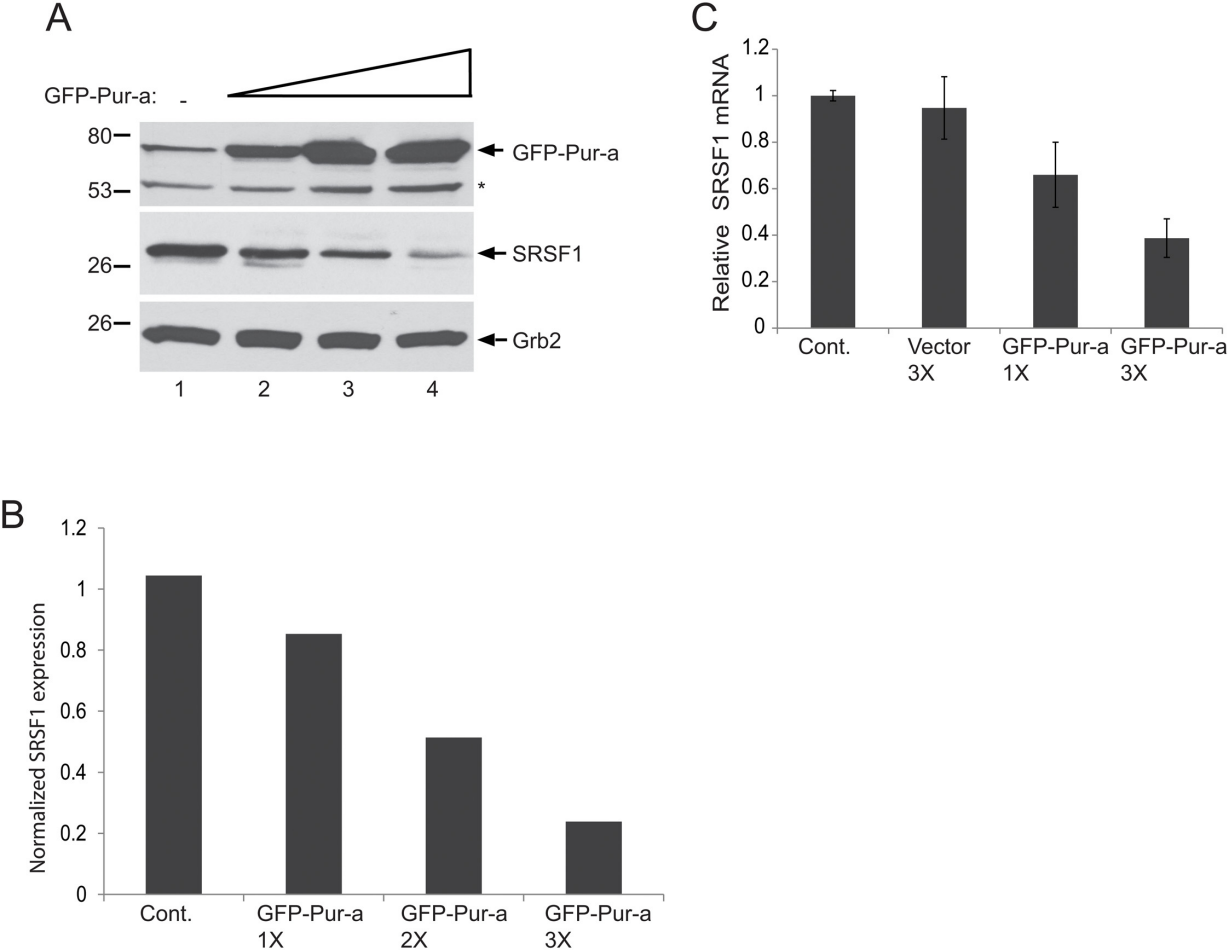
Dose dependent inhibition of SRSF1 by Pur-alpha in glial cells. T98G cells were transfected with increasing concentrations of pEGFP-Pur-alpha plasmid (1X, 2X, and 3X) and whole cell protein extracts were prepared at 48hr post-transfection. Expression of SRSF1 and Pur-alpha were detected by Western blotting using anti-Pur-alpha and anti-SRSF1 antibodies. Grb2 was also probed in the same blots as an internal loading control. B. Signal intensities of SRSF1 expression in panel A were normalized to Grb2 levels and are shown as a bar graph. C. T98G cells were transfected with increasing concentrations of an expression plasmid encoding GFP-Pur-alpha. Total RNA was extracted and expression levels of SRSF1 mRNA transcripts were determined by Q-RT-PCR. mRNA levels of SRSF1 were normalized and are presented relative to change in relation to the control as a bar graph. All experiments were carried out in triplicate.

The observed attenuation of SRSF1-mediated suppression of JCV transcription and T-antigen expression have prompt us to hypothesize that Pur-alpha could possess its function by directly targeting and suppressing SRSF1 gene expression. In order to test this, we examined the impact of Pur-alpha overexpression on endogenous levels of SRSF1 in glial cells. Primary human fetal astrocytes (PHFA) were transfected with increasing concentrations of an expression vector encoding GFP-Pur-alpha. Total DNA concentration per condition was kept equal by co-transfecting with GFP control vector. As shown in Fig 3A and 3B, Pur-alpha expression in PHFA cells caused a dose dependent reduction in the endogenous levels of SRSF1 protein, suggesting that Pur-alpha exhibits a negative effect directly on SRSF1 gene expression in glial cells. To gain more insight into the Pur-alpha mediated suppression of SRSF1 protein levels, we analyzed SRSF1 mRNA expression by Q-RT-PCR. In parallel with protein lysates, total RNA from the same experiments presented in panel A were also prepared in parallel and analyzed by Q-RT-PCR for the detection and quantification of SRSF1 mRNA transcripts. As shown in Fig 3C, ectopic expression of Pur-alpha caused a dose dependent reduction in the levels of SRSF1 transcripts, suggestion that Pur-alpha exhibits its function by directly suppressing expression of the SRSF1 gene.

### Inverse expression of Pur-alpha and SRSF1 in Pur-alpha (-/-) MEF cells

**Fig 4:**
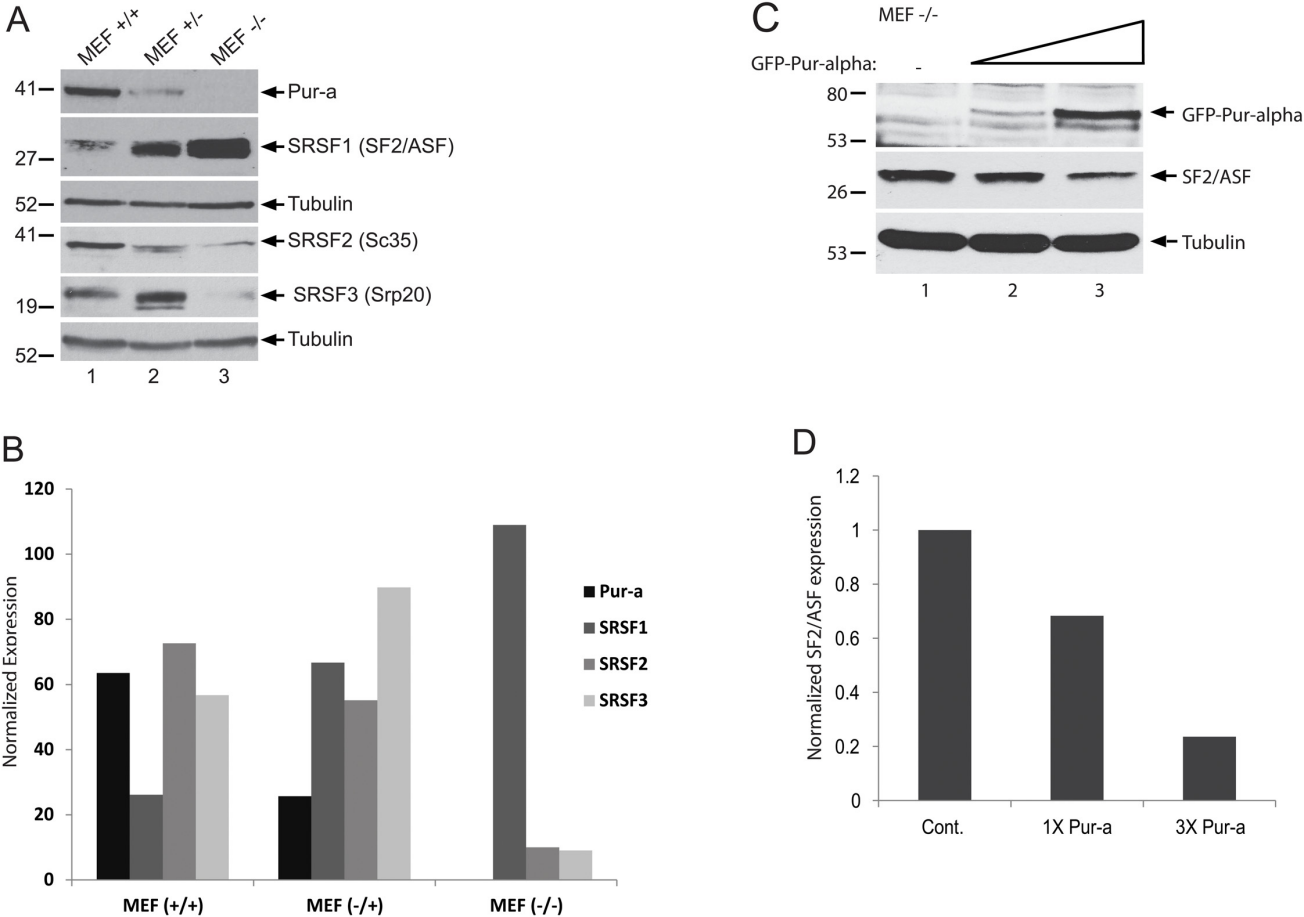
Inverse correlation of Pur-alpha and SRSF1 protein levels in Pur-alpha (-/-) MEF cells. **A.** Lysates were prepared from mouse embryonic fibroblast (MEF) cultures obtained from wild type mice (MEF+/+), mice heterozygous for targeted disruption of the PURA gene (MEF+/-), or mice with homozygous deletion in PURA (MEF-/-). Whole cell protein extracts were analyzed by Western blotting for the expression of Pur-alpha, SRSF1, SRSF2, and SRSF3. Tubulin was probed in the same blots as an internal loading control. B. Band intensities of Pur-alpha, SRSF1, SRSF2, and SRSF3 in panel A were determined, normalized to tubulin, and are shown as a bar graph. C. Pur-alpha MEF-/- cells were transfected with an expression plasmid encoding GFP-Pur-alpha in increasing concentrations (1X and 3X). Whole cell protein lysates were prepared at 48 hrs post-transfection and examined by Western blotting for the detection of SRSF1 and Pur-alpha levels. Tubulin was probed in the same blots as an internal loading control. Band intensities of SRSF1 levels in panel C were quantified, normalized to tubulin, and are shown as a bar graph.

In order to gain more insight into the biochemical expression and functional interaction between Pur-alpha and SRSF1, we analyzed protein lysates prepared from mouse embryonic fibroblast (MEF) cultures obtained from wild type mice (MEF+/+), mice heterozygous for targeted disruption of the PURA gene (MEF+/-), or mice with homozygous deletion in PURA (MEF-/-) by Western blotting. As shown in Fig 4A and 4B, wild type MEF +/+ cells showed strong pur-alpha expression with considerably lower but detectable levels of SRSF1 (panel A, lane 1). Interestingly, the ratio of Pur-alpha/SRSF1 proteins was shifted and significantly reduced in MEF +/- cells, and SRSF1 levels were elevated nearly 5 fold in MEF -/- cells (panel A, compare lanes 1, 2, and 3). These observations suggest that SRSF1 expression shows an inverse correlation with Pur-alpha expression in MEF cells. In addition to SRSF1, SRSF2 and SRSF3 expression levels were also measured and compared to SRSF1 and Pur-alpha in the MEF cells. Interestingly, unlike SRSF1, SRSF2 expression levels showed a positive correlation with the expression of Pur-alpha. On the other hand, although we observed a significant induction in SRSF3 levels in MEF +/- cells compared to MEF +/+, its expression was barely detectable in lysates from MEF -/- cells. To further confirm the inverse correlation in the expression levels of SRSF1 and Pur-alpha, MEF -/- cells were transfected with increasing concentrations of an expression plasmid encoding GFP-Pur-alpha. As shown in Fig 4C and 4D, ectopic expression and restoration of Pur-alpha levels in Pur-alpha MEF -/- cells caused a significant reduction in SRSF1 levels in a dose dependent manner. These observations have revealed an inverse relationship between Pur-alpha and SRSF1 expression levels.

### Effect of Pur-alpha and SRSF1 on JCV genomic DNA replication

**Fig 5:**
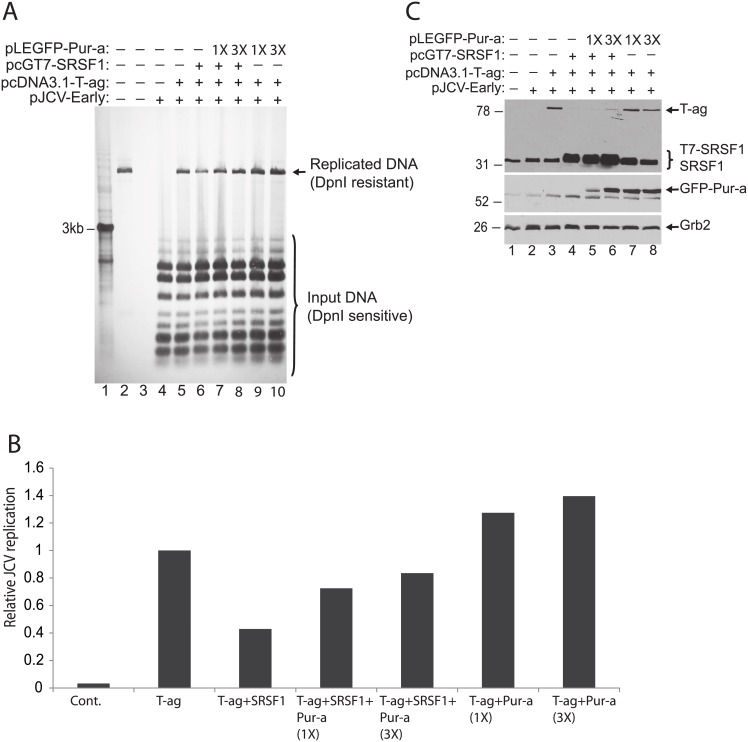
Pur-alpha blocks SRSF1 induced inhibition of JCV DNA replication. A. DpnI replication assay in PHFA cells transfected with replication competent JCV plasmid DNA and expression plasmids encoding JCV T-antigen, T7-tagged SRSF1, and GFP-tagged Pur-alpha as indicated. DNA and protein lysates were prepared from cells harvested 7 days post-transfection. DpnI digestion of DNA was performed to distinguish newly replicated DNA from the transfected input plasmid DNA. B. Results from DpnI assay in Panel A are presented as a bar graph. C. Western blot analysis of protein lysates harvested in parallel to the DpnI assay in Panel A for the expression of T7-tagged SRSF1 and GFP-tagged Pur-alpha. Tubulin was probed in the same blots as loading control.

To further demonstrate the functional interaction between Pur-alpha and SRSF1 and their impact on JCV reactivation, we performed a DpnI replication assay in the presence of SRSF1 and increasing concentrations of Pur-alpha. PHFA cells were transfected with a reporter plasmid containing the JCV promoter and origin of DNA replication (pJCV-Early). Cells were also transiently transfected with expression plasmids encoding T7-tagged SRSF1, T-antigen, and increasing concentrations of GFP-tagged P-alpha. At 72 hrs post-transfections, low molecular weight DNAs were extracted and processed by DpnI/Southern blotting assay for the detection of newly replicated plasmid DNA. As shown in Fig 5A and 5B, newly replicated DNA was detected in PHFA cells transfected with a plasmid containing the JCV promoter and origin of DNA replication (pJCV- Early) as well as plasmids expressing T-antigen, T7-SRSF1, and increasing concentrations of GFP-Pur-alpha. Digestion with DpnI, which cuts non-methylated input plasmid DNA, but not the methylated newly replicated DNA, revealed the level of DNA replication in the cells, as shown in Panel A and quantitated as fold change over T-antigen-induced replication as shown in Panel B. SRSF1 decreased JCV DNA replication by nearly 60%, while this suppression was inhibited by the addition of Pur-alpha. In the absence of SRSF1, Pur-alpha enhanced JCV replication by 40% over that of T-antigen alone. Expression levels of transfected constructs were also confirmed by Western blotting (panel C) in whole cell protein lysates prepared in parallel to DNA samples from the same experiments presented in panel A.

### Detection of Pur-alpha and SRSF1 in PML oligodendrocyte inclusion bodies

**Fig 6:**
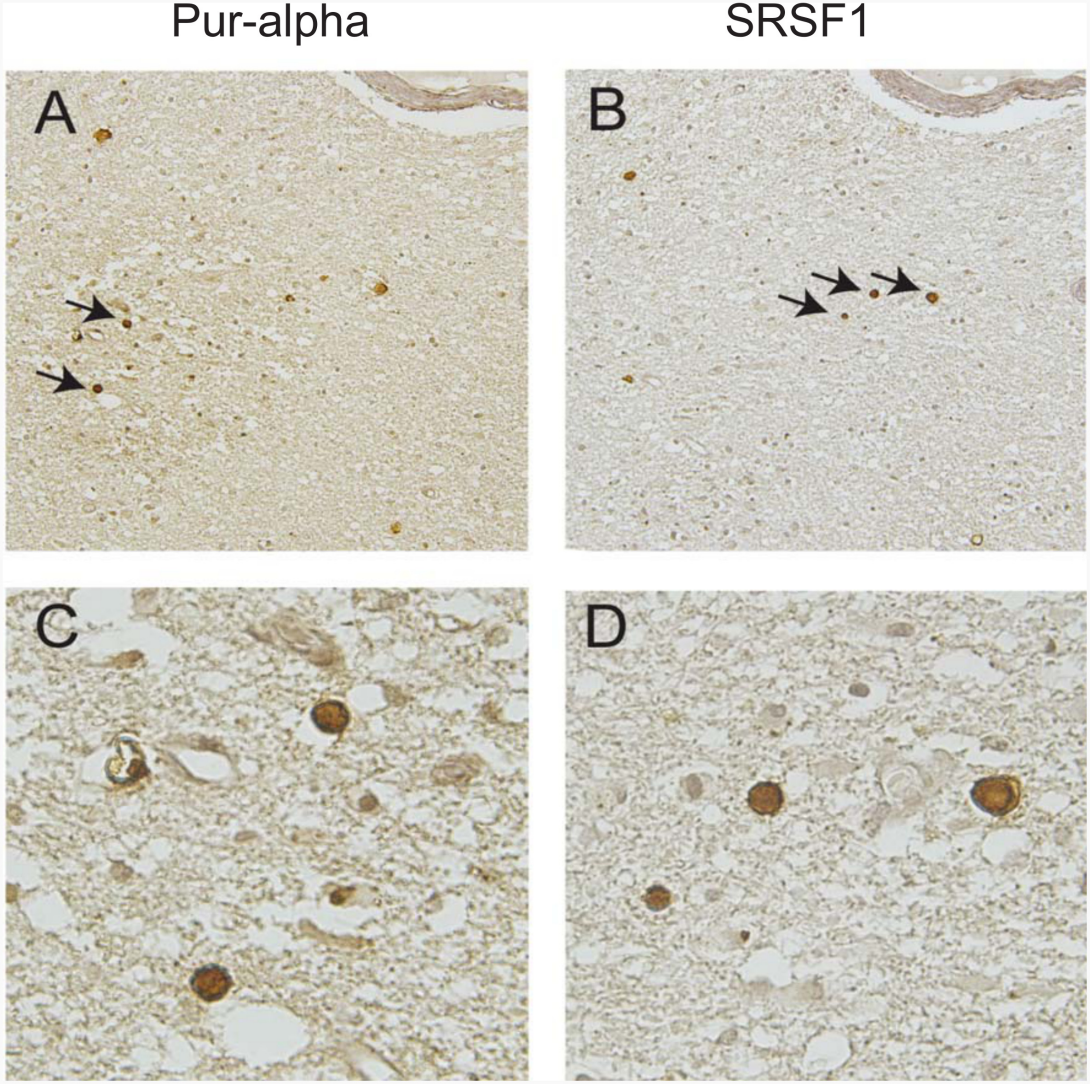
Detection of Pur-alpha and SRSF1 in oligodendrocyte inclusion bodies of PML-AIDS. Immunohistochemistry was performed on serial sections of AIDS-PML tissue samples from three cases of HIV-1-associated PML obtained from Dr. Susan Morgello, Manhattan HIV Brain Bank (MHBB) with antibodies to detect Pur-alpha (A and C) and SF2 (B and D). Both proteins were detected in the nuclei of oligodendrocyte inclusion bodies, the site of JC virus replication in PML. Arrows in panels A and B highlight the cells shown in panels C and D at higher magnification. Panels A and B, original magnification x200. Panels C and D, original magnification x400.

In order to examine the relevance to PML of this inverse functional relationship between Pur-alpha and SRSF1, immunohistochemistry was performed on serial sections of AIDS-PML brain tissue samples obtained from Dr. Susan Morgello, Manhattan HIV Brain Bank, NNTC. Serial tissue sections of PML brain lesions were immunolabeled with antibodies to Pur-alpha and SRSF1 (Fig 6). As indicated by the arrows, both Pur-alpha and SRSF1 expressions were detected in oligodendrocytes containing inclusion bodies, which are the pathological hallmark of PML and the sites of replication for JC virus. Interestingly, inclusion bodies were typically positive for either Pur-alpha or SRSF1, though some cells appeared to be positive for both proteins. These data suggest that Pur-alpha and SRSF1 are expressed in JCV infected oligodendrocytes during active viral replication, and may impact viral reactivation.

## Discussion

Viruses evade host cells and must gain access to the host cellular machinery for their own gene expression and genomic DNA replication, as well as for completion of the viral life cycle. The efficiency and productivity of viral replication depends on a delicate balance among host factors which are positive and negative regulators of viral gene expression and DNA replication. SRSF1 is an important host gene controlling JC virus gene expression in glial cells [19–21,32]. We have recently showed that SRSF1 expression is regulated by immune signaling, and can be induced by soluble immune mediators (cytokines and chemokines) leading to transcriptional suppression of JC virus [33]. More importantly, SRSF1 induction is required for the suppression of JCV gene expression and viral replication mediated by neuroimmune conditioning. Here, our results from a series of biochemical assays suggested that SRSF1 protein and mRNA expression are significantly suppressed by Pur-alpha in glial cells. Since Pur-alpha shows a positive impact on JCV gene expression and viral replication, these observations suggest the possibility of a functional interaction between Pur-alpha and SRSF1 in neuroimmune suppression of JC virus. Whether, similar to SRSF1, Pur-alpha gene expression is also regulated by soluble immune mediators and associated with neuroimmune suppression of JCV remains to be further studied.

SRSF1-mediated suppression of JCV is mediated by a dual action mechanism targeting both JCV transcription and T-antigen mRNA splicing. It has been demonstrated that SRSF1 can directly target the JCV promoter and suppress both early and late gene transcription in glial cells [19,20]. In addition to a transcriptional blockage of viral promoter activity, SRSF1 is also capable of targeting JCV -early pre-mRNA splicing and maturation leading to suppression of T-antigen expression [19,34]. This dual-action suppression of JCV gene expression puts SRSF1 in a special position in regulation of JCV reactivation and the viral lytic infection cycle. On the other hand, contrary to SRSF1, Pur-alpha has been shown to be an activator of JCV transcription [29,31]. The observed suppression of SRSF1 and increased expression of T-antigen protein levels may suggest a novel role of Pur-alpha in regulation of JCV early gene splicing in glial cells. It is possible that Pur-alpha could enhance T-antigen mRNA splicing by reducing the protein levels of SRSF1.

Immunohistochemical evaluation of P-alpha and SRSF1 expression in PML brain lesions has revealed that these two proteins are both detected in oligodendrocyte inclusion bodies. Interestingly, these inclusion bodies were typically positive either for SRSF1 or Pur-alpha suggesting that the two proteins could be expressed during different stages of the viral lytic cycle. One can postulate that SRSF1 is possibly induced and expressed during viral latency as well as early in the replication cycle of JCV as an innate host response to viral infection. Pur-alpha gene could be activated and expressed by immediate early viral proteins, including T-antigen, during the mid and late stages of the viral life cycle leading to excessive late gene expression for the production of viral capsid proteins as well as for replication of the viral genome. The exact mechanism and functional interaction between Pur-alpha and SRSF1 in regulation of JCV reactivation leading to the productive lytic viral life cycle remains to be further elucidated.

In summary, we have demonstrated an antagonistic relationship between cellular SRSF1 and Pur-alpha proteins in the regulation of JCV. A series of molecular and biochemical assays have demonstrated that Pur-alpha is capable of relieving the negative pressure on JCV gene expression mediated by SRSF1 leading to initiation of viral reactivation and genomic replication. These observations have suggested an important functional interaction between these two proteins, and may suggest a novel mechanism of JCV reactivation leading to development PML. Understanding the contribution of these two proteins, and the timing of their expression, may lead to new therapeutic targets for the treatment of PML.

## Materials and Methods

### Ethics statement

Cultures of primary human fetal astrocytes (PHFA) were prepared from human fetal brain tissue obtained under approval of the Temple University Institutional Review Board (IRB).

### Cell lines and cultures

Primary human fetal astrocytic cells (PHFA) were cultured from fetal brain and provided by the Comprehensive NeuroAIDS Core facility (CNAC) in the Department of Neuroscience at the Lewis Katz School of Medicine at Temple University. Human derived T98G glioblastoma cell lines were obtained from American Type Culture Collection (ATCC) and were cultured in Dulbecco's Modified Eagle's Medium (DMEM) supplemented with 10% heat-inactivated fetal bovine serum (FBS) and antibiotics (penicillin/streptomycin, 100 μg/ml). Both PHFA and T98G cells were maintained at 37°C in a humidified atmosphere with 5% CO2. Mouse embryonic fibroblast (MEF) cultures obtained from wild type mice (MEF+/+), mice heterozygous for targeted disruption of the PURA gene (MEF+/-), or mice with homozygous deletion in PURA (MEF-/-) were described previously [35,36].

### Plasmid constructs

The luciferase reporter construct pLuc-JCV-Early was made by blunt end cloning of the full-length JCV Mad-1 non-coding control region (NCCR) into the SmaI site immediately upstream of the luciferase gene in the plasmid pGL3 (Promega, Madison WI) as described previously [37]. pCGT7-SRSF1 expression plasmid, kindly provided by Javier F. Cáceres (Medical Research Council Human Genetics Unit, Western General Hospital, Edinburgh EH4 2XU, Scotland, United Kingdom) was described previously [19,38]. JCV T-antigen (JCV Mad-1 5013–2603, NC_001699.1), was cloned into vector pcDNA3.1(+) at EcoRI restriction enzyme site as previously described [39]. pEGFP-C1-Pur-alpha was produced by cloning of Pur-alpha from pGEX-1lambdaT-Pur-alpha [40] into the Kpn1/BamHI sites of pEGFP-C1 (Clontech).

### Luciferase reporter assay

T98G cells were plated in 6 well tissue culture dishes and transiently transfected with pLuc-JCV-Early reporter plasmid and expression vectors encoding T-antigen, T7-tagged SRSF1, and GFP-tagged pur-alpha at various combinations. All transfections were normalized with the addition of empty vector DNA. At 48 hours post-transfection, cells were extracted and lysed using reporter lysis buffer for the luciferase reporter system provided by the manufacturer (Promega, USA). Luciferase activities of samples were determined as described by the manufacturer. The luciferase activities were then corrected for protein concentrations and normalized to the basal level of transcription, allowing for the determination of relative luciferase activity and fold change over basal transcription level.

### Real Time PCR

RT-PCR amplification and quantification of SRSF1 transcripts were performed as described previously [41]. Briefly, total RNA was isolated using the RNeasy kit (Qiagen, Valencia, CA). 500 ng of RNA was reverse-transcribed and subjected to RT- PCR amplification. RT-PCR was carried out with SYBR Green I master mix on a LightCycler 480 (Roche Applied Science). All PCR reactions were performed in triplicate. Primer sequences were: SRSF1-forward 5′-TCT CTG GAC TGC CTC CAA GT-3′ and reverse 5′-GGC TTC TGC TAC GAC TAC GG-3′.

### Non-radioactive Southern blotting and DpnI assay

Low molecular weight DNA purified from JCV-infected PHFA cells were digested with DpnI and BamHI enzymes, separated on 1% agarose gels and then transferred to nylon membranes. Replicated viral DNA was visualized using a DIG-High Prime DNA Labeling and Detection Kit according to the Manufacturer’s instructions (Roche). The whole JCV Mad1 genome was linearized by BamH1 digestion, labeled with DIG, and used as probe for hybridization of the membranes.

### Immunohistochemistry for Pur-alpha and SRSF1

Formalin-fixed, paraffin-embedded sections of HIV-PML brain tissues were kindly provided by Dr. Susan Morgello, Manhattan HIV Brain Bank, NNTC, sectioned at 4μM in thickness. Immunohistochemistry was performed using the avidin–biotin–peroxidase complex system (Vectastain Elite ABC Peroxidase Kit, Vector Laboratories, Inc., Burlingame, CA). After deparaffinization with xylene, rehydration, non-enzymatic antigen retrieval was performed in 0.01M citrate (pH 6.0) for 30 min at 95°C followed by cooling for 20 min. All sections were then quenched for endogenous peroxidase in methanol/3% H2O2 for 20 min. After blocking with serum/BSA, primary antibodies were added and incubated overnight in a humidified chamber. Sections were then incubated with the appropriate biotinylated secondary antibody followed by detection with DAB (0.02% diaminobenzidine and 0.005% hydrogen peroxide), counterstaining with hematoxylin, and mounting with Permount.

### Antibodies

Antibodies utilized for immunoblotting and immunohistochemistry include monoclonal mouse anti-Pur-alpha antibody developed in the laboratory of Dr. Edward M. Johnson (clone 10B12), mouse monoclonal SV40 T-Antigen (pAb416, Calbiochem), mouse monoclonal anti-SRSF1 (Clone 96, Invitrogen), mouse monoclonal anti-SRSF2 (Ab11826, Abcam), and rabbit polyclonal anti-SRSF3 (HPA056981, Sigma-Aldrich). Mouse monoclonal anti-GRB2 antibody (clone 81, BD Transduction Laboratories, Franklin Lakes, NJ) or anti-tubulin antibody (clone B512, Abcam, Boston, MA) were used as loading controls for Western blotting.
